# Evaluation of long-lasting microbial larvicide for malaria vector control in Kenya

**DOI:** 10.1186/s12936-016-1626-6

**Published:** 2016-12-01

**Authors:** Yaw A. Afrane, Nixon G. Mweresa, Christine L. Wanjala, Thomas M. Gilbreath III, Guofa Zhou, Ming-Chieh Lee, Andrew K. Githeko, Guiyun Yan

**Affiliations:** 1 Department of Medical Microbiology, College of Health Sciences, University of Ghana, Accra, Ghana; 2 Climate and Human Health Research Unit, Centre for Global Health Research, Kenya Medical Research Institute, Kisumu, Kenya; 3 Program in Public Health, College of Health Sciences, University of California, Irvine, CA 92697 USA; 4 Department of Medical Laboratory Sciences, Masinde Muliro University of Science and Technology, Kakamega, Kenya

**Keywords:** Larval control, Biological larvicide, Long-lasting larvicide, *Bacillus thuringiensis israelensis*, *Bacillus sphaericus*

## Abstract

**Background:**

Outdoor malaria transmission is becoming an increasingly important problem in malaria control in Africa. Larval control is a promising intervention as it can target both indoor and outdoor biting mosquitoes. However, the currently available biolarvicide formulations have a short effective duration, and consequently larval control incurs a high operational expense due to the requirement for frequent re-treatment of larval habitats. Formulations of biolarvicides with long-lasting effects is highly desired. A recently developed FourStar® slow-release briquet formulation of *Bacillus thuringiensis israelensis* and *Bacillus sphaericus* was evaluated to test its efficacy on malaria vectors.

**Methods:**

The study evaluated FourStar™ briquets 180-days formulation under semi-natural and natural conditions to test their efficacy in reducing the mosquito population in western Kenya. The semi-natural habitats used the formulation dissolved in rainwater with appropriate concentrations, and second-instar larvae of *Anopheles gambiae* were introduced and the number of surviving larvae and pupae produced was recorded daily as the outcome. The briquets formulation was then tested in natural habitats for efficacy on pupal productivity reduction in highland and lowland sites in western Kenya. The formulation was finally tested for efficacy in reducing adult mosquito populations in randomized clusters in western Kenya highland.

**Results:**

In semi-natural conditions, the FourStar™ briquets 180-days formulation completely inhibited mosquito pupal production in the first 3 months, and then reduced pupal productivity by 87–98% (P < 0.001) 4–6 months after application. In natural habitats, during the first 2 months no pupae were detected from any of the treated habitats in highland sites, and *Anopheles* spp. pupal density was reduced by 60–90% in the next 3–5 months (P < 0.001). In the lowland site, pupal productivity reduction was 100% in the first 3 months, and 75–90% in the next 4–5 months (P < 0.001). The randomized cluster trial found that the application of the briquets formulation reduced mean densities of indoor-biting mosquitoes by 76–82% (P < 0.001) and by 67–75% (P < 0.001) for outdoor-biting mosquitoes.

**Conclusion:**

This study demonstrated that long-lasting biological larviciding was effective in reducing pupal productivity of larval habitats, and reducing indoor and outdoor resting mosquitoes. The study suggests that long-lasting microbial larviciding may be a promising complementary malaria vector control tool and warrants further large-scale evaluation.

## Background

Malaria continues to be a leading cause of death and morbidity in sub-Saharan Africa despite the massive scale-up of long-lasting impregnated nets (LLINs) and indoor residual spraying (IRS) interventions within the last decade [1]. Despite overall success in reducing malaria prevalence and incidence, there is evidence of limited impact and a resurgence of clinical malaria in parts of sub-Saharan Africa [2, 3]. There is increasing evidence for shifts in vector biting behaviour from night biting to early biting, and from indoor biting to outdoor biting, and for vector species composition change from previously predominantly indoor-biting *Anopheles gambiae* to predominantly *Anopheles arabiensis* or other not-yet-identified vector species that prefer to bite and rest outdoors [3–5]. However, the front-line malaria vector control tools only target indoor-resting and indoor-biting vectors and do not kill the mosquitoes that bite and rest outdoors, leading to a surge in residual malaria transmission [6, 7]. Residual transmission of malaria is becoming an important public health problem in Africa [6]. Additionally, LLIN and IRS control tools are insecticide-based, and resistance to insecticides continues to spread across Africa [8, 9]. There is a pressing need to include interventions that could tackle residual malaria transmission in the present malaria control paradigm and to develop viable insecticide resistance management strategies to help malaria control and elimination efforts in sub-Saharan Africa.

Larval source management (LSM), including habitat modification, manipulation and larviciding, has historically been the main focus of mosquito control programmes in many parts of the world, which helped to control and eliminate malaria in countries such as the USA, Canada, in some European countries, and Brazil [10–12]. However, Africa has seen little LSM application due to the perception of high operational cost and complexity [13]. LSM has been shown in several studies in Africa to be effective in reducing the abundance of malaria mosquito larvae, adults and transmission [14, 15]. LSM offers the dual benefits of reducing numbers of outdoor- and indoor-biting mosquitoes [13] and, therefore, may be valuable in tackling prevalent residual malaria transmission that is a major challenge in malaria control in Africa. The Cochrane review by Tusting et al. [16] concluded that “in Africa and Asia, LSM is another policy option, alongside LLINs and IRS, for reducing malaria morbidity in both urban and rural areas where a sufficient proportion of larval habitats can be targeted”. One important question to be addressed is whether LSM is feasible and cost effective in parts of rural Africa where larval habitats are more extensive and malaria transmission intensity is high.

The currently available biolarvicide formulations have a short, effective duration (7–10 days), and they have been shown to be effective in reducing malaria vector abundance and transmission [17–20]. However, larval control may incur a high operation expense if frequent re-treatment of larval habitats is required. Formulations of biological larvicides that have long-lasting effects would reduce application costs because habitats would require less frequent re-treatment. Recently, slow-release formulations of microbial larvicides have been developed and approved by United States Environmental Protection Agency for field mosquito control. The present study evaluated the effectiveness of a slow-release microbial larvicide based on *Bacillus thuringiensis israelensis* and *Bacillus sphaericus* in reducing habitat productivity and adult malaria vector abundance in Kenya. The potential advantages of using *Bacillus sphaericus* and *Bacillus thuringiensis israelensis* in one larvicide formulation include reduced rate of resistance development to bacterial toxins, mosquito-specific killing and no known negative impact on non-target organisms [13].

## Methods

### Long-lasting microbial larvicide tested

The present study examined a United States Environmental Protection Agency approved, slow-release FourStar™ briquets 180-days formulation manufactured by Adapco Inc [21]. The 30 g formulation consists of 6% by weight of *Bacillus sphaericus* (Bs) Serotype H5a5b strain 2362, 1% by weight of *Bacillus thuringiensis* sub-species *israelensis*, (Bti) strain BMP 144 and other ingredients that make the formulation to release slowly the bacterial toxins.

### Effect of FourStar™ briquets 180-days formulation under semi-natural conditions

To test the efficacy and effective duration of the slow-release microbial larvicide formulation in killing malaria mosquitoes under semi-natural conditions, the 30-g FourStar™ briquets 180-days formulation was placed in 560 L of rainwater in a 1000-L container. This amount of water was calculated based on the recommended dose of one FourStar™ briquet in a 9.3-sq metres of surface area. The briquet was dissolved, stored in the container and covered with insect-proof net, as the stock solution. Three stock solutions were prepared. Microcosms were prepared in a plastic basin (30 cm diameter and 20 cm deep) using 2 kg soil from the rural community in Kisian village, western Kenya and 2 L of stock solution. Control microcosms used 2 kg soil and rainwater without any larvicide.

Sixty-second-instar field collected *An. gambiae* larvae were introduced to each microcosm basin each month for a period of 6 months. At each time point, ten microcosms were used, including five basins with microbial larvicide and five control basins without any larvicide. Water in basins was replenished from the stock solution or rainwater to compensate for water loss due to evaporation. The number of surviving larvae and pupae were monitored and recorded daily. Pupae productivity was used as a proxy measure for adult mosquito emergence, and it was used as the key measurement of the effectiveness of microbial larvicide here [22].

### Testing FourStar™ briquets 180-days formulation under field conditions

**Fig. 1:**
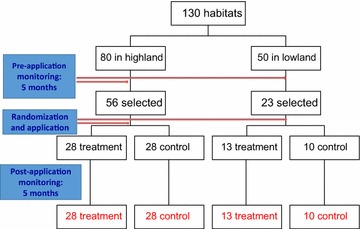
Sample size flow chart for the field testing of slow-release FourStar™ Briquets 180 days microbial larvicide in a cohort of aquatic habitats in western Kenya

After the promising result in the semi-natural conditions, further experiments were conducted to test the efficacy and effective duration under the field conditions. Eighty *An. gambiae* larval habitats were selected in a highland site of Iguhu and 50 in a lowland site of Kisian in western Kenya to determine their stability and productivity, for a period of 5 months from September 2009 to January 2010. Habitat stability was the time period during which larval habitats remained aquatic to support larval development. Habitat productivity was the number of pupae produced in a habitat. These habitats were monitored every 2 weeks for the entire duration of the study. Sampling was done to determine the larval and pupal density per habitat. After the period of monitoring, stable and productive habitats were selected for either treatment or no treatment with the briquet formulation. Fifty-six habitats in the highland site of Iguhu and 23 habitats in the lowland site of Kisian were stable and productive, so they were randomized into intervention and non-intervention habitats, stratified by highland and lowland sites. There were 28 habitats in the intervention arm, and 28 in the control arm in the highland site, and 13 in the intervention arm and ten in the control arm in the lowland site (Fig. 1). Because of an extremely large amount of rainfall due to El Nino event in western Kenya from end of January to April 2010, the FourStar™ briquets 180-days microbial larvicide was applied in larval habitats of intervention arm in May 2010, using the recommended dosage of one 30-g briquet per 9.3-m^2^ of surface area. Habitats in the control arm were not treated by microbial larvicide. All breeding habitats were monitored monthly from June to October 2010 to determine pupal productivity. Rainfall data were obtained from local meteorological stations for both sites.

### Effect of FourStar™ briquets 180-days formulation on indoor-resting malaria vector abundance

**Fig. 2:**
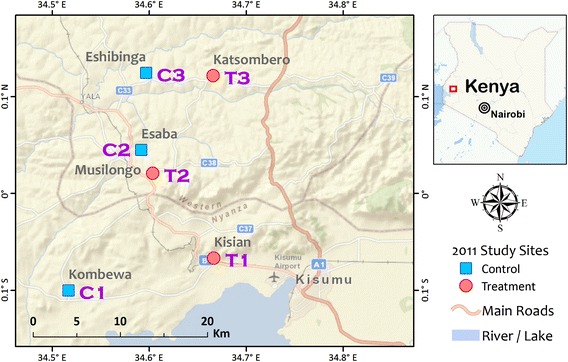
A map of study sites used for randomized cluster study of slow-release FourStar™ Briquets 180 days microbial larvicide. The six site were located in three epidemiological regions, including malaria endemic lowland region (Kisian and Kombewa), epidemic-prone highland-fringe region (Esaba and Musilongo), and epidemic highland region (Eshibinga and Katsombero). The area size of each site is 2 x 2 km^2^

Six 2 × 2 km^2^ sites from lowland, highland fringe and highland regions in western Kenya were used to determine the effect of slow-release microbial larvicide on adult malaria vector abundance reduction (Fig. 2). Two sites were located in malaria-endemic lowland (Kisian and Kombewa, 1150–1200 m above sea level) [3]. Two sites were in malaria-endemic highland fringe region (Esaba and Musilongo, elevation 1380–1480 m), and two sites (Eshibinga and Katsombero) were located in malaria epidemic-prone highland (elevation 1500–1650 m) [3]. Within each region, one site was randomly assigned as an intervention site and the other as a no-intervention control site by coin toss. A baseline adult mosquito density survey was conducted in all sites using the pyrethrum spray catch (PSC) method in 60 randomly selected houses in January 2011. All larval habitats within the study sites were sampled for larval distribution and abundance at baseline. All larval habitats within the intervention sites were treated with FourStar™ briquets 180-days formulation. Briquets were placed with respect to larval habitat sizes, according to manufacturer’s recommendations for each briquet. No habitats in the control sites were treated by the larvicide. Larvicide was applied in February 2011 during the dry season, and evaluation of the intervention was conducted from March to June 2011. The primary endpoint was adult mosquito abundance. Adult mosquito population abundance was monitored weekly in 20 randomly selected houses within each site using the PSC method. Because of residual mosquito killing and repellent effect of pyrethrum used in PSC collection, the houses were rotated on weekly basis, and thus in each site 60 houses were sampled. To minimize the migration effects of mosquitoes from neighbouring larval habitats outside the 2 × 2 km^2^ intervention area, all 60 houses selected for evaluation were located within 500 m from the edge of the study area.

### Effect of FourStar™ briquets 180-days formulation on indoor- and outdoor-resting malaria vector abundance in a randomized trial

**Fig. 3:**
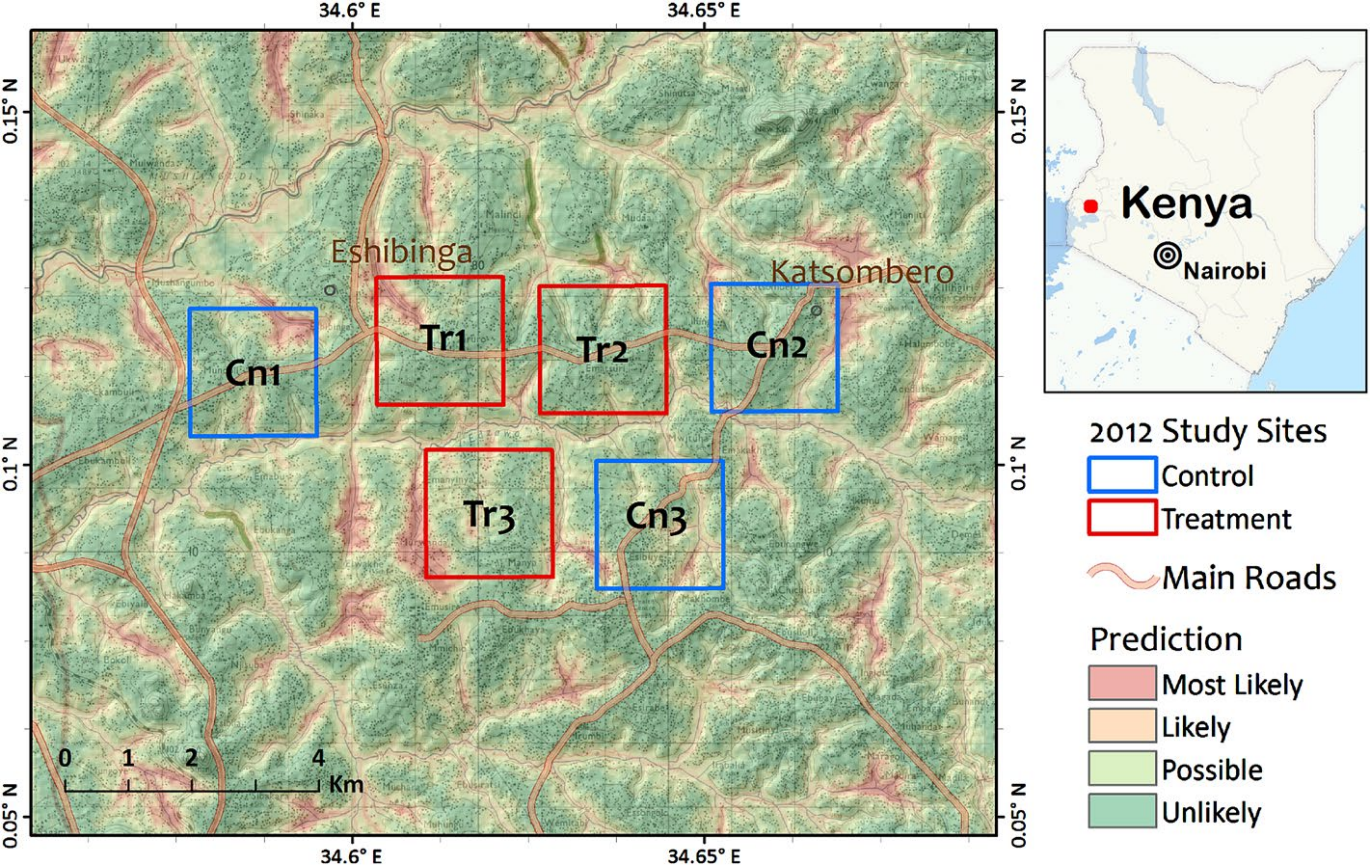
A map of study sites in the highland area used for randomized paired cluster study of slow-release microbial larvicide

To test the hypothesis that the long-lasting microbial larvicides can reduce the abundance of adult vectors inside houses and outdoor, a randomized, paired-cluster design was undertaken in 2012 with six 2 × 2 km^2^ sites selected in Katsombero located in the highland area of western Kenya where breeding habitats were generally clustered around streams and valley bottom [23, 24] (Fig. 3). The six selected sites were randomized into intervention and control sites. Larval and adult mosquito populations were monitored in these sites at baseline in January 2012. Sixty houses were randomly selected in the sites and indoor and outdoor biting mosquito density was estimated using CDC light traps. In the intervention sites breeding habitats were then treated with the *Bs/Bti* briquet formulation whilst habitats in the control sites received no treatment. The briquets were applied in all larval breeding sites at the middle of the dry season (February) and just before the rainy season (March) to reduce the dilution effect of rainfall. Mosquito abundance indoor and outdoor was monitored using the CDC light traps. Each week, 20 of the 60 houses selected were sampled in rotation. CDC light traps were hanged indoors and outdoors in five houses per night for four nights per week. Because our objective was to compare the extent of indoor and outdoor biting in areas treated and not treated with the long-lasting larvicides, the vector abundance monitoring method with the use of CDC light traps was appropriate.

### Data analysis

In the experiments to test the efficacy and duration of efficacy of the briquets in mosquito control under semi-natural environment, *t* test was done to determine the statistical differences in the mean number of pupae emerging from the treated and untreated microcosms. For experiments to determine the efficacy of the briquets in pupal productivity control in natural habitats, analysis of variance (ANOVA) with repeated measures was done to determine the statistical differences between the intervention and control habitats. For the studies to determine the effect of the briquet on mosquito densities inside and outside houses, densities of mosquito per house per night was calculated in the intervention and control sites, and ANOVA with repeated measures was used to determine the statistical significance. Numbers of *An. gambiae* s.l. and *An. funestus* mosquitoes were pooled due to very low *An. funestus* abundance.

## Results

### FourStar™ briquets 180-days formulation under semi-natural conditions

**Fig. 4:**
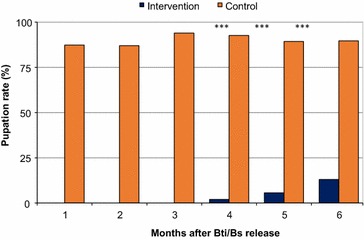
Effect of slow-release FourStar™ Briquets 180 days microbial larvicide formulation on pupation rate of *Anopheles gambiae* mosquitoes in semi-natural conditions

In the semi-natural microcosm experiment, no pupae emerged from any treated microcosms during the first 3 months, whilst a pupation rate of 89.4% was found in the untreated control microcosms. In months 4, 5 and 6, pupation rate was 2.0, 5.7 and 13.0% in the treated microcosms, respectively, whilst that of the untreated microcosms were 92.7, 89.3 and 89.7%, respectively (*t* test, P < 0.0001 for all comparisons) (Fig. 4). This result suggests that the FourStar™ briquets 180-days formulation reduced pupal productivity of *An. gambiae* mosquitoes in semi-natural microcosms for up to 6 months by at least 85.5%.

### Effects of FourStar™ briquets 180-days formulation on pupal productivity in field conditions

**Fig. 5:**
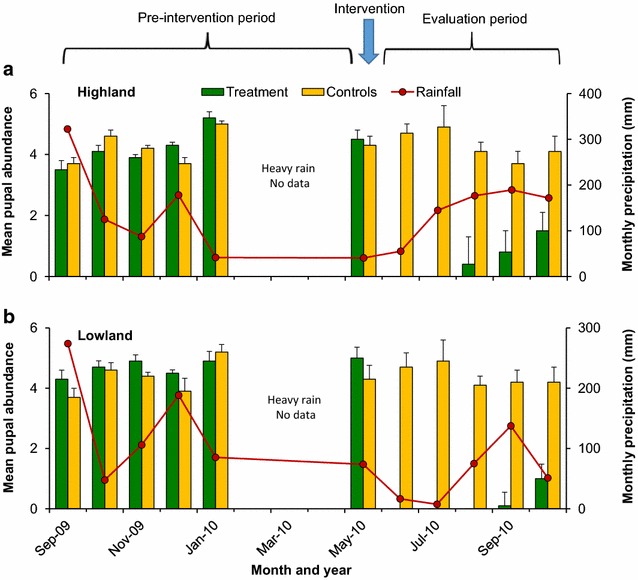
Effect of slow-release FourStar™ Briquets 180 days microbial larvicide formulation on pupal productivity of malaria vectors in a cohort of natural larval habitats in the highland (**a**) and lowland (**b**) of western Kenya

During the study period the average monthly rainfall was 139.2 mm in the highland site and 17.1 mm in the lowland sites. Pre-intervention rainfall was 150.7 and 17.2 mm in the highland and lowland, respectively, whilst post intervention average monthly rainfall was 147.4 and 16.7 mm (Fig. 5a, b).

In the highland site, pupal productivity between intervention and control habitats was similar during the 5-month pre-intervention period (4.20 vs 4.24 pupae per dip, F_1,8_ = 0.01, P > 0.05; Fig. 5a). In the first 2 months after the larviciding application, no pupae were detected from any of the treated habitats in the highland areas whereas the average productivity of control habitats was 4.8 pupae per dip (Fig. 5a). From months 3 to 5, the average pupae density in the intervention habitats was 0.73 whilst that in the control habitats was 3.9, leading to a relative reduction of 87.4% in pupal productivity (F_1,8_ = 110.79, P < 0.0001).

Similar to findings in the highland sites, the pupae productivity in the habitats in the lowland site in the intervention and control arm were similar (4.60 vs 4.36 pupae per dip, F_1,8_ = 1.07, P > 0.05). Pupal productivity was 0 during the first three months after microbial larvicide application, and 4.6 in the control habitats. In months 4 and 5, pupal productivity was 0.55 in the intervention habitats whereas 4.2 pupae were found in the control habitats, a reduction of 95.4% (F_1,8_ = 276.5, P < 0.0001; Fig. 5b).

### Effect of FourStar™ briquets 180-days formulation on indoor-resting malaria vectors

**Fig. 6:**
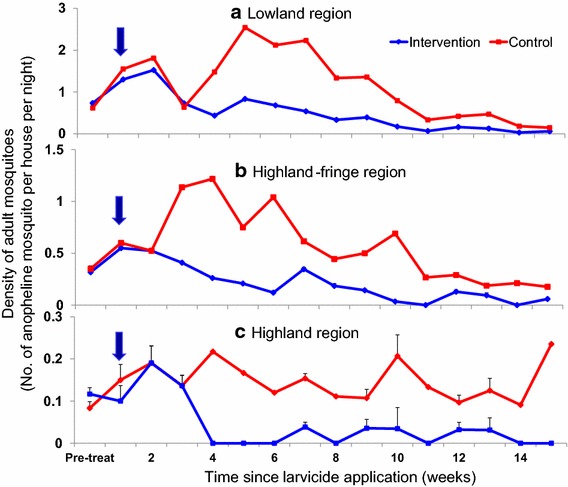
Effect of slow-release microbial larvicide formulation on indoor malaria vector abundance in lowland (**a**), highland-fringe (**b**) and highland (**c**) area of western Kenya

Prior to larviciding, anopheline adult mosquito density collected indoors by PSC was comparable for each of the three areas. Generally, the impact of larviciding on adult mosquitoes was observed two to three weeks after larviciding. The application of the FourStar™ briquets formulation caused a reduction of 60–85% in the density of adult malaria vectors in the next 14 weeks in the lowland (F_1,22_ = 9.64, P < 0.005; Fig. 6a). Larviciding impact on mosquito density started to decay 9 weeks after larviciding application. In the highland fringe region, application of FourStar™ briquets reduced adult mosquito density by 65–80% in the following 14 weeks (F_1,22_ = 15.53, P < 0.001), and the effect of larvicide started to decay 10 weeks after larviciding (Fig. 6b). In the highland, larvicide exhibited the longest period of effectiveness, a reduction of 76–90% in adult density was found 14 weeks after larviciding (F_1,22_ = 76.92, P < 0.0001; Fig. 6c). A total of 1812 mosquitoes were collected for this experiment, comprising of 88.9% (1610) *An. gambiae* s.l., 4.9% (89) *An. funestus* and 6.2% (113) *Culex* spp.

### Effect of FourStar™ briquets 180-days formulation on indoor- and outdoor-resting malaria vector abundance in a randomized trial

**Fig. 7:**
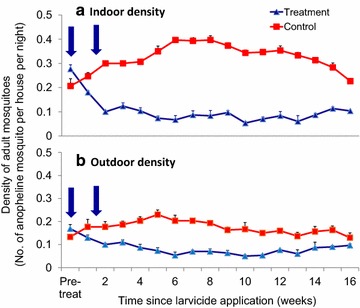
Impact of slow-release FourStar™ Briquets 180 days microbial larvicide formulation on the densities of adult malaria vector collected indoors (**a**) and outdoors (**b**) in the highland area

Pre-intervention vector density within the intervention sites was 0.28 female mosquitoes per house per night compared to 0.21 in the control sites. During the first 3 months post-application of the briquets, the density of adult malaria vectors inside residential houses within the intervention sites was decreased by 76–82% compared to the untreated control sites (0.09 vs 0.33; F_1,32_ = 131.87, P < 0.0001; Fig. 7a). The density of malaria vectors caught outdoors was reduced by 67–75% (0.08 vs 0.18; F_1,32_ = 76.03, P < 0.0001; Fig. 7b). Thus, the slow-release microbial larviciding significantly reduced indoor- and outdoor-biting mosquitoes for at least 3 months. A total of 3325 mosquitoes were sampled for this study, comprising of 84.8% (2821) *An. gambiae* s.l., 5.4% (179) *An. funestus* with the rest being *Culex* spp. 9.7% (325).

## Discussion

In the present study, a new slow-release microbial larvicide formulation based on *Bacillus sphaericus* and *Bacillus thuringiensis israelensis* was tested against malaria vectors in semi-natural microcosms and natural conditions for both pupal productivity and adult vector densities in rural communities in western Kenya. There was a 100% reduction in pupation rate in the first 3 months, and decay of larviciding efficacy to 85.5% reduction of pupation rate 5 months after larviciding application in semi-natural microcosms. In natural habitats, efficacy of the briquets in suppressing pupal productivity of malaria vectors was demonstrated in malaria epidemic highlands and endemic lowlands. The slow-release microbial larvicide was effective in inhibiting pupal productivity by 100% in the first 2–3 months, and by 87.4–95.4% within 3–5 months of larviciding application. Cluster randomized studies demonstrated a reduction of 76–82% in indoor vector abundance and 67–75% in outdoor vector abundance within the 14 weeks monitored. These results showed that slow-release microbial larvicide formulation presents a promising malaria vector control tool.

When the slow-release formulation was applied in the field, bacterial toxins took several days to build up the concentration to a level lethal to mosquito larvae or to inhibit larval metamorphosis to pupae. Although the larviciding effect was not instant, the formulation we tested had a longer effect in comparison to other commercial microbial larvicides previously examined for malaria vector control, e.g. [16, 25, 26]. In the study sites in western Kenya the effective duration of the briquets lasted 2–3 months, before larviciding efficacy started to decay. The highland sites exhibited faster decay rate than the lowland sites (Fig. 5), most likely due to more intense rainfall in the highlands than the lowland sites. Rainfall could have diluted the briquets that were placed in the larval habitats and therefore caused a decay in larviciding effect. Additionally, some briquets sunk into the mud beneath the habitat bottom and were not available to effectively release any bacterial toxins. These factors may explain the decay of larviciding efficacy after 3 months of use in the field.

In the randomized cluster experiments, it was shown that application of slow-release briquet formulations caused reduction of indoor and outdoor adult malaria vector abundance by 60–85% for a period of 3 months. The formulation we evaluated was labeled as “180-days formulation”, but our evaluation was conducted for only 3 months, and whether the briquettes remain effective for a longer period of time needs future field testing. Longer effective duration would reduce the frequency of habitat re-treatment and thus help to reduce operational costs [20]. It is unknown whether reduction in the density of adult vectors will translate into reduction in the incidence of clinical malaria or malaria infections. Because outdoor malaria transmission represents one of the most important challenges in malaria control in Africa [6], slow-release briquet formulations warrant further evaluation of the impact on clinical malaria in multiple countries. Important questions should be addressed prior to large-scale testing, such as, is the dry season the optimal timing for larviciding application; what is the efficient strategy to identify stable habitats for larviciding; how frequently should larvicides be applied; should larvicide dosage be adjusted for larval habitats with different vegetation coverage and water depth; would resistance to bacterial toxins evolve rapidly in mosquito populations due to imperfect killing of larvae by larvicides; what proportion of larval habitats must be treated to achieve targeted level of reduction in malarial incidence; and under what ecological setting is long-lasting larviciding cost-effective? Mathematical modelling, in combination with field ecological experimentation can help address these questions.

## Conclusion

In summary, there is a growing recognition that integrated malaria vector management is needed to control and eliminate malaria [27–30]. Larval source management could be employed to complement the use of LLINs and IRS to target residual transmission [28–30]. Microbial larvicides with long residual activity such as the *Bacillus sphaericus* and *Bacillus thuringiensis israelensis* briquet formulation tested in the present study would be a valuable addition to the toolbox for integrated malaria vector management.
